# Symptom clusters and help-seeking behaviours during neutropenia after autologous haematopoietic stem cell transplantation: A qualitative study to inform oncology nursing support

**DOI:** 10.1371/journal.pone.0361023

**Published:** 2026-10-15

**Authors:** Lili Jiang, Junxue Hua, Fang Teng, Ting Xu, Hongyun Cao, Jingfen Zhou

**Affiliations:** Hematology Department, The Affiliated Hospital of Jiangnan University, Wuxi, Jiangsu, China; Stanford University, UNITED STATES OF AMERICA

## Abstract

**Purpose:**

To explore how adult autologous haematopoietic stem cell transplantation recipients experience and appraise symptom constellations during neutropenia, and how these appraisals shape help-seeking and interactions with clinicians.

**Methods:**

A qualitative descriptive study was conducted in an inpatient haematopoietic stem cell transplantation unit in Wuxi, China. Adults aged 18 years or older who had undergone autologous transplantation and were neutropenic were purposively sampled to maximise variation. Semi-structured interviews were audio-recorded, transcribed verbatim, and analysed using reflexive thematic analysis.

**Results:**

Twenty-two participants were interviewed between post-transplant day + 5 and +18. Participants described symptoms as interlocking constellations rather than isolated events. They differentiated expected treatment-related toxicities from possible deterioration by attending to symptom trajectories, including worsening intensity, new symptom combinations, or symptoms that felt different from the previous day. Fever-related patterns usually prompted immediate escalation. Help-seeking ranged from prompt reporting to watchful waiting, self-management followed by reporting, or withholding. These responses were shaped by uncertainty, perceived legitimacy of concerns, and reluctance to bother staff during protective isolation.

**Conclusions:**

Symptom constellations and symptom trajectories shaped urgency appraisal and help-seeking during inpatient neutropenia after autologous haematopoietic stem cell transplantation. Cluster-informed assessment and escalation coaching may support timelier reporting and safer supportive care in protective isolation.

## Introduction

Haematopoietic stem cell transplantation (HSCT) can be curative for many haematological diseases, yet the early post-transplant neutropenic phase is marked by profound physiological vulnerability, strict infection-prevention routines, and a high likelihood of treatment-related toxicities [1]. Even with prophylactic antimicrobials and protective isolation, infection remains a major threat, while patients simultaneously contend with burdensome symptoms such as fatigue, mucositis-related pain, gastrointestinal disturbance, sleep disruption, and anxiety—often in an environment that restricts usual sources of comfort and informal support [2,3].

Importantly, these experiences are seldom “single-symptom” problems. Symptom cluster research suggests that co-occurring symptoms may share mechanisms, amplify one another, and shape what patients notice and prioritise day to day [4]. In HSCT populations, prior work has identified relatively stable clusters (e.g., fatigue–sleep–emotional symptoms) and more variable gastrointestinal or mucocutaneous groupings, as well as the possibility that certain sentinel symptoms reorganise patients’ attention by signalling deterioration [5]. Conceptual models further emphasise that people interpret interrelated symptom experiences holistically; these interpretations influence coping strategies, disclosure, and care-seeking choices [6].

Help-seeking for cancer treatment complications has therefore become a central concern, particularly because normalising symptoms as “expected,” uncertainty about urgency, and prior experiences with healthcare can contribute to delayed escalation [7–9]. However, much of the existing evidence focuses on ambulatory contexts (e.g., home-based chemotherapy) rather than the distinct situation of hospitalised HSCT recipients in protective isolation—where close monitoring coexists with limited spontaneous contact, complex treatment expectations, and social dynamics that may inhibit “bothering” staff for non-alarm concerns [7,10,11]. Qualitative studies describe distress, loss of autonomy, and uncertainty during isolation, yet rarely integrate a symptom-cluster lens with patients’ real-time escalation decisions during high-risk inpatient neutropenia [12,13]. To address this gap, our qualitative descriptive study explored how adult HSCT recipients experienced and interpreted co-occurring symptoms during the neutropenic phase, how they distinguished expected toxicities from warning patterns, and how these appraisals shaped their help-seeking behaviours and interactions with nurses and physicians.

## Methods

### Study design

This study used a qualitative descriptive design with semi-structured, in-depth interviews to explore how adult haematopoietic stem cell transplantation (HSCT) recipients experienced co-occurring symptoms and how they appraised these symptoms and sought help during the neutropenic phase. This study is reported in accordance with the Consolidated Criteria for Reporting Qualitative Research (COREQ). A completed 32-item COREQ checklist is provided as Supporting Information (S1 Checklist).

### Setting

The study was conducted in the inpatient HSCT unit of a tertiary hospital in Wuxi, China, where recipients were routinely cared for in protective isolation rooms during the early post-transplant neutropenic phase. Standard supportive care in the unit included close monitoring of vital signs and laboratory indices, antimicrobial prophylaxis as clinically indicated, and nursing-led symptom assessment and education consistent with unit protocols.

### Participants and recruitment

A purposive, maximum-variation sampling strategy was applied to capture diverse symptom experiences and help-seeking patterns, with variation sought in sex, age, and post-transplant day. Participants were eligible if they: (1) were aged ≥18 years; (2) had undergone autologous haematopoietic stem cell transplantation (HSCT); (3) were in the neutropenic phase at the time of interview, operationally defined as an absolute neutrophil count (ANC) <0.5 × 10^9^/L and/or receiving protective isolation for neutropenia according to unit practice; (4) were able to communicate in Mandarin; and (5) provided written informed consent. Exclusion criteria were: (1) impaired consciousness or severe cognitive or psychiatric symptoms precluding meaningful participation; (2) clinical instability, such as severe respiratory distress or haemodynamic instability, as judged by the treating team; or (3) refusal or withdrawal of consent.

For descriptive reporting, the **duration of neutropenia at the time of interview** was calculated as the number of days from the first documented ANC < 0.5 × 10^9^/L to the date of the qualitative interview.

Recruitment occurred between June 2024 and December 2025. Potentially eligible patients were first identified by clinical staff and subsequently approached by the research interviewer, who explained the study, answered questions, and obtained written informed consent. Twenty-four eligible patients were approached for the formal study; two declined participation because of fatigue or a preference not to participate in an interview. Twenty-two participants were therefore enrolled and completed the study, with no withdrawals after consent.

Sample adequacy was evaluated using the Information Power framework proposed by Malterud et al. [14], which considers the study aim, sample specificity, theoretical background, quality of dialogue, and analysis strategy. The final sample of 22 participants was considered to provide sufficient information power for several reasons. First, the study had a focused aim centred on how adults undergoing autologous HSCT experienced and appraised co-occurring symptoms and made help-seeking decisions during the clearly bounded neutropenic phase. Second, the sample was highly specific to this clinical context: all participants had undergone autologous HSCT, were experiencing neutropenia, and were cared for in protective isolation, while maximum-variation sampling across sex, age, and post-transplant day captured clinically relevant experiential diversity. Third, the interview guide was informed by established literature on symptom clustering, symptom appraisal, and help-seeking, providing a coherent conceptual background without constraining inductive interpretation. Fourth, the face-to-face interviews were sufficiently in-depth, typically lasting 30–60 minutes, and contemporaneous field notes provided additional contextual detail on symptom experiences and decision-making. Finally, the iterative reflexive thematic analysis involved repeated within- and across-case comparison, and successive interviews increasingly elaborated and refined existing analytic patterns rather than introducing substantively different dimensions relevant to the study aims. Taken together, these considerations supported the adequacy of 22 participants for the present analysis.

### Research team and relationship with participants

The study team comprised six registered nurses from the Hematology Department of The Affiliated Hospital of Jiangnan University. Interviews were conducted by Lili Jiang, RN, a female registered nurse with eight years of clinical experience in haematology and haematopoietic stem cell transplantation (HSCT) care. Before the study commenced, she had received structured training in qualitative research methods, semi-structured interviewing, reflexive interviewing techniques, and reflexive thematic analysis. She was not responsible for participants’ direct clinical care during the study and had no established therapeutic relationship with participants before recruitment. Before each interview, participants were informed of the interviewer’s professional background, her role as a member of the research team, the purpose of the study, and the voluntary nature of participation. The interviewer maintained a reflexive journal throughout data collection to document interview dynamics, assumptions, and potential influences of her clinical background on data interpretation.

### Data collection

Data were collected through face-to-face, semi-structured interviews conducted in participants’ protective-isolation rooms to minimise burden and align with infection-control requirements. Only the participant and the interviewer were present during the interview; no family members or clinical staff participated in the interview sessions. An interview guide was developed based on the study aims and sensitised by prior literature on symptom clustering and symptom appraisal/help-seeking. Before formal data collection, the interview guide was pilot-tested with two autologous HSCT recipients who met the eligibility criteria but were not included in the final analytic sample. The pilot interviews indicated that the overall interview structure was feasible; minor wording and sequencing adjustments were made to improve clarity and reduce participant burden. Interviews explored: (1) the full range of symptoms experienced during neutropenia; (2) which symptoms tended to occur together and how participants described their co-occurrence; (3) perceived “warning signs” versus “expected” symptoms; (4) decision thresholds and triggers for seeking help; (5) preferred routes and timing of help-seeking (e.g., calling the nurse immediately vs waiting for rounds); (6) self-management attempts before seeking help; and (7) facilitators and barriers (e.g., isolation environment, fear of infection, prior experiences, perceived staff responsiveness, and communication concerns).

Interviews were conducted face-to-face in Mandarin by Lili Jiang. Interviews typically lasted 30–60 minutes, were audio-recorded with permission, and were supplemented by contemporaneous field notes capturing context (e.g., interruptions, fatigue, emotional cues) and reflexive observations. No repeat interviews were conducted.

Audio recordings were transcribed verbatim. To support English-language dissemination, transcripts (and quotations selected for reporting) were translated into English using a forward-translation approach by a bilingual team member, then reviewed against the original Mandarin transcript by a second bilingual reviewer to ensure semantic equivalence; discrepancies were resolved through discussion within the research team.

### Data analysis

Analysis proceeded iteratively alongside data collection using reflexive thematic analysis. Two researchers, Lili Jiang and Junxue Hua, were actively involved in coding and theme development. Lili Jiang led the initial coding of the complete dataset, while Junxue Hua reviewed coded extracts and emerging analytic patterns at regular intervals. Rather than using inter-coder agreement as a reliability criterion, differences in interpretation were discussed reflexively to explore alternative meanings and challenge clinically driven assumptions. Transcripts were first read repeatedly for familiarisation, and initial impressions were documented in analytic memos. Meaning units relevant to symptom experiences, symptom co-occurrence, appraisal processes, and help-seeking behaviours were coded inductively. Codes were then compared and organised into code clusters, subthemes, and candidate themes, with particular attention to recurring symptom co-occurrence patterns and the decision logic linking symptom interpretation with help-seeking actions. Candidate themes were repeatedly reviewed against the full dataset, including negative and deviant cases, before being refined, defined, and named. Representative quotations were selected to illustrate the analytic interpretation.

Qualitative data management, including coding, retrieval, and maintenance of the analytic audit trail, was supported by NVivo version 15.3.0 (Lumivero).

### Rigour and trustworthiness

Trustworthiness was supported through iterative data collection and analysis, contemporaneous field notes, reflexive journaling, regular analytic meetings, active consideration of discrepant or deviant cases, and maintenance of an audit trail documenting interview-guide development, coding decisions, code-cluster development, theme-development memos, and reflexive notes. Formal member checking was not undertaken, and interview transcripts or final themes were not returned to participants for validation. Consistent with the reflexive thematic analytic approach, credibility was instead supported through sustained engagement with the dataset, reflexive memoing, repeated within- and across-case comparison, discussion of alternative interpretations, and close examination of disconfirming evidence. Dependability and confirmability were supported by the audit trail and team-based analytic discussion, while transferability was facilitated by detailed description of the clinical context, protective-isolation environment, and participant characteristics.

### Researcher reflexivity

The research team included nurses with clinical experience in haematology and HSCT care. This background provided familiarity with neutropenia, treatment-related toxicities, fever escalation, and routine symptom surveillance, which sensitised the researchers to clinically meaningful changes in participants’ symptom narratives. However, the team also recognised that this clinical positioning could privilege a safety- and escalation-oriented interpretation of help-seeking, whereby delayed reporting might initially be viewed as insufficient symptom knowledge or failure to act on clinical advice. Reflexive journaling and analytic memoing were therefore used throughout data collection and analysis to identify and examine these assumptions. During team debriefing, particular attention was paid to participants’ own explanations for waiting, rechecking symptoms, seeking “proof,” postponing disclosure until routine rounds, or withholding concerns because they perceived staff as busy. These accounts prompted the team to move beyond a simple dichotomy of “timely” versus “delayed” reporting and to interpret help-seeking as a negotiated process in which clinical uncertainty was weighed against perceived legitimacy, anticipated staff burden, and the social consequences of asking for help. This reflexive process contributed directly to the development of the theme “Help-seeking was ‘negotiated’: appraisal, thresholds, and the need for ‘proof’.” Reflexive notes and alternative interpretations were revisited during analytic meetings, and discrepant cases were actively considered to ensure that clinical assumptions did not override participants’ meanings.

### Ethical considerations

Ethical approval for this study was obtained from the Medical Ethics Committee of The Affiliated Hospital of Jiangnan University (Approval No. LS2024516). All participants provided written informed consent prior to participation. Confidentiality was ensured by de-identifying transcripts and storing recordings/transcripts on password-protected devices accessible only to the research team. Given participants’ clinical vulnerability during neutropenia, interviews were paused or discontinued if participants experienced fatigue or distress; if participants disclosed symptoms suggestive of acute deterioration, the interviewer followed a predefined safety procedure by promptly notifying the responsible clinical staff in accordance with unit policy.

### Patient and public involvement

Patients and the public were not involved in the study design, recruitment, conduct, analysis, or dissemination plans.

## Results

### Participant characteristics

A total of 22 HSCT recipients in the neutropenic phase participated in the interviews (12 women and 10 men; median age, 44 years; range, 19–66 years). All participants had undergone autologous HSCT. The underlying diagnoses were lymphoma (n = 8) and multiple myeloma (n = 14). At the time of interview, participants were between post-transplant day + 5 and +18 and had experienced neutropenia for a median of **6 days (range, 2–13 days)**. All participants were cared for in protective isolation rooms. Five participants had experienced at least one febrile episode since transplantation, and 18 reported substantial disruption of sleep and daily routines. **Participant characteristics are summarised in Table 1**.

**Table 1:** Participant characteristics.

Characteristic	Value
Participants, n	22
Age, median (range), years	44 (19–66)
Sex, n	Female 12; Male 10
Transplant type, n	Autologous 22
Primary diagnosis, n	Lymphoma 8; Multiple myeloma 14
Post-transplant day at interview	+5 to +18
Duration of neutropenia at interview, median (range), days	6 (2–13)
Protective isolation at interview	22/22
Any febrile episode, n	5
Sleep disruption reported, n	18

### Overview of symptom clusters and help-seeking patterns

Participants rarely talked about symptoms as isolated events. Instead, they described symptoms clustering and cascading, and help-seeking as a stepwise decision process shaped by perceived severity, uncertainty, time of day, and perceptions of staff workload. Across the dataset, symptom experiences converged into three core clusters—(1) gastrointestinal–oral discomfort–fatigue, (2) fever–chills–pain, and (3) sleep disruption–anxiety–isolation—while help-seeking behaviours were expressed through four pathways: immediate reporting, watchful waiting, self-management then reporting, and minimisation/withholding. Importantly, participants’ accounts suggested that the same symptom could trigger different pathways depending on co-occurring symptoms, prior experiences, and contextual cues (e.g., night-time, isolation, recent fever episode).

### Theme 1. A “baseline cluster” dominated everyday functioning: gastrointestinal–oral discomfort–fatigue

#### 1.1. A chain reaction that disrupted intake and activity.

Participants commonly described oral/throat discomfort (often framed as mouth sores, burning pain, or dryness) co-occurring with nausea, taste changes, early satiety, and profound fatigue. This constellation was perceived as a “baseline” burden that reduced oral intake and led to weakness and low mood.

“When my mouth hurts, I don’t want to eat; when I don’t eat, I feel weak; when I’m weak, everything feels worse—including the nausea. It’s like a loop.” (P07)

#### 1.2. “Expected but still unbearable”: normalisation delayed escalation.

Although participants often recognised these symptoms as expected during neutropenia, the label of “expected” sometimes **lowered help-seeking urgency**, even when symptoms were severe enough to impair nutrition and sleep.

“I thought this is part of transplant, so I tried to endure. I only called when I realised I had taken almost nothing for the whole day.” (P14)

#### 1.3. Self-management as the first-line strategy.

Many participants described trying small, incremental coping strategies before reporting: sipping water, choosing soft foods, pacing, distraction, or tolerating short periods of discomfort. Help was sought when symptoms became persistent, clustered more tightly, or crossed a personal threshold (e.g., “cannot swallow,” “cannot keep anything down,” “too weak to get up”).

“At first I tried to manage it myself—small sips, resting. But when the diarrhoea and nausea came together, I felt I couldn’t control it anymore.” (P09)

### Theme 2. The “alarm cluster” (fever–chills–pain) triggered rapid escalation and low thresholds for reporting

#### 2.1. Fever as a sentinel symptom.

Fever was repeatedly framed as a high-risk sign during neutropenia and was often associated with chills, body aches, headache, and a sense of impending danger.

“Once the temperature goes up, it’s not a small thing in this period. I press the call bell immediately.” (P03)

#### 2.2. Rapid escalation scripts and expectations of response.

Participants described a clear escalation script: measure temperature → inform nurse → expect rapid assessment, cultures/labs, and intensified monitoring. This script provided psychological reassurance and reduced uncertainty.

“When they come quickly and explain what they’re checking, I feel I’m not alone with the fear.” (P19)

#### 2.3. Hypervigilance after a febrile episode.

After experiencing fever once, some participants became more vigilant and reported bodily sensations earlier (“feeling hot,” “feeling cold,” “heart racing”), reflecting a shift in appraisal thresholds.

“After I had fever once, even a small chill makes me nervous. I’m afraid I’ll miss the early sign.” (P05)

### Theme 3. Nights amplified the sleep–anxiety–isolation cluster and reshaped help-seeking

#### 3.1. Night-time symptom amplification.

Participants described night-time as a period when symptoms and worries intensified—insomnia, palpitations, ruminative thoughts, and loneliness.

“In the daytime you can distract yourself. At night it’s quiet, and your mind keeps turning. Even small discomfort feels bigger.” (P11)

#### 3.2. Emotional symptoms were often minimised or withheld.

Unlike fever, emotional distress was frequently judged as “not serious enough” to report. Many hesitated to contact staff for anxiety or loneliness, especially when they perceived staff as busy.

“I didn’t want to disturb them for ‘just anxiety’. But it kept me awake and made everything feel worse.” (P02)

#### 3.3. Help-seeking for reassurance rather than treatment.

When participants did seek help for this cluster, they often emphasised the need for reassurance, information, or presence rather than medication.

“I called not because I needed medicine, but because I needed someone to tell me whether this is normal.” (P08)

### Theme 4. Help-seeking was “negotiated”: appraisal, thresholds, and the need for “proof”

#### 4.1. Building a case before calling.

For ambiguous symptoms (dizziness, mild breathlessness, abdominal discomfort), participants often sought “proof” first: rechecking temperature, waiting to see if it persists, comparing to prior days.

“I observe first. If it passes quickly, maybe it’s nothing. If it repeats, then I feel I have a reason to ask.” (P16)

#### 4.2. Timing strategies: wait for rounds vs interrupt now.

Many participants preferred to wait for routine rounds to avoid interrupting, unless symptoms belonged to the alarm cluster or became unbearable.

“If it’s not fever, I wait for rounds. But if it’s night and I feel something is wrong, I won’t wait.” (P20)

### Theme 5. “Watching the numbers”: lab values, monitors, and uncertainty as drivers of help-seeking

Participants often linked symptom appraisal to objective indicators—temperature readings, blood counts, infusion rates, and clinicians’ language about “low immunity.” However, many felt uncertain about what specific values meant for symptom urgency, creating a gap that shaped help-seeking.

“They tell me my neutrophils are very low, but I don’t know what symptoms are ‘danger’ and which ones are ‘normal’. That uncertainty is stressful.” (P06)

Some participants described relying heavily on staff interpretation of numbers, which influenced when they sought help—particularly when symptoms were mild but anxiety was high.

“If someone explains what today’s results mean, I feel calmer and I don’t overthink every sensation.” (P12)

### Theme 6. Communication in isolation: practical barriers and social considerations shaped reporting

#### 6.1. Practical friction: PPE, distance, and “small problems”.

Participants described barriers unique to isolation—limited spontaneous contact, the need to use the call bell, delays in face-to-face reassurance, and concern about bothering staff.

“In isolation you can’t just ask casually. You have to press the bell. For small symptoms, I hesitate.” (P10)

#### 6.2. Social calibration: not wanting to be seen as “difficult”.

Patients often calibrated requests based on perceived staff workload and a desire to be a “good patient,” sometimes delaying reporting of non-alarm symptoms.

“I see they are busy. I don’t want them to think I complain too much, so I hold back unless it’s serious.” (P17)

### Theme 7. Family involvement shifted but did not disappear: “remote co-management”

Despite physical separation, family remained influential through phone/video contact. Families encouraged reporting, helped interpret symptoms, or increased anxiety by emphasising worst-case possibilities.

“My family tells me to call the nurse immediately. Sometimes that helps me act, but sometimes it makes me more scared.” (P04)

Participants differed in whether family contact was comforting or stressful, and this shaped help-seeking thresholds—especially at night.

### Theme 8. What participants wanted: concrete guidance to support timely and appropriate help-seeking

Across interviews, participants articulated practical needs that could improve symptom management and communication:

#### 8.1. Clear escalation criteria (what requires immediate reporting vs observation).

“If they give a simple rule—what to report immediately—I would feel less uncertain.” (P13)

#### 8.2. A night-time plan (how to seek reassurance and manage insomnia/anxiety).

“Nights are hardest. If there was a plan for what to do when you can’t sleep and feel panic, it would help.” (P21)

#### 8.3. Symptom–cluster framing rather than single-symptom advice.

“It’s not one symptom. It comes as a package. Advice should match that.” (P07)

#### 8.4. Permission to report emotional distress without feeling “unreasonable”.

“If someone told me it’s okay to ask for help even for anxiety, I would not wait until it becomes unbearable.” (P02)

## Discussion

### Interpretation of the main findings

This study clarifies how hospitalised adult HSCT recipients in protective isolation make sense of co-occurring symptoms during the neutropenic phase, and how these appraisals shape help-seeking and interaction patterns with nurses and physicians. Our findings extend the symptom-management literature by showing that symptom experiences were rarely isolated “single problems.” Instead, participants interpreted symptom constellations (e.g., mucosal–gastrointestinal discomfort co-occurring with fatigue, sleep disturbance, and emotional strain) as meaningful patterns that required constant recalibration of what was “expected” versus what signalled potential deterioration.

This pattern-based reasoning is consistent with growing evidence that HSCT recipients experience high, multi-symptom burden during acute transplant care and that symptom intensity tends to peak around the first 1–2 weeks post-transplant, when neutropenia and treatment toxicities converge. In inpatient settings, daily electronic patient-reported outcome (ePRO) studies have documented both the breadth of symptoms and the clinical value of identifying symptom worsening early—supporting the idea that symptom experience during acute transplantation is dynamic and requires frequent reassessment.

### Co-occurring symptoms as “clusters” and the practical problem of attribution

Participants’ accounts are consistent with the broader cancer symptom‐cluster literature and with emerging HSCT work showing that physical and psychological symptoms frequently co-occur and reinforce one another rather than appearing as discrete, independent problems. For example, in a cross-sectional study of allo-HSCT recipients, fatigue, insomnia, depression, anxiety, and stress were all highly prevalent and intercorrelated, underscoring the tendency for “biobehavioural” symptoms to travel together post-transplant [15]. Similarly, large survivor studies in transplantation have highlighted that high fatigue is closely associated with anxiety, depression, and sleep problems, and—critically for our focus on escalation—many recipients report barriers to discussing fatigue and related symptoms with their care team, suggesting that co-occurrence alone does not guarantee timely disclosure [16]. Beyond mapping co-occurrence, recent HSCT symptom research has begun to propose “sentinel symptom” approaches, in which certain symptoms may act as early indicators within a cluster and therefore offer pragmatic targets for proactive assessment and intervention [4]. However, our data add an important nuance for the inpatient neutropenic phase in protective isolation: symptom clustering is not merely a statistical pattern but a cognitive and communicative task that patients must perform under uncertainty. Participants described persistent difficulty attributing symptoms to (a) “expected” conditioning-related toxicities, (b) isolation-related distress and disrupted routines, or (c) emerging warning patterns requiring escalation; notably, the same symptom (e.g., nausea, oral pain, fatigue) could feel “normal” on one day but signal danger on another depending on co-occurrence (new combinations), trajectory (worsening over hours/days), and perceived controllability (response—or lack of response—to routine self-management or prescribed supportive care). This attribution work shaped not only whether patients called for help, but also how they framed concerns (as tolerable side effects versus “something different”), how much they downplayed symptoms to avoid “bothering” staff, and how willing they were to persist in uncertainty when objective monitoring (vital signs/labs) appeared reassuring—highlighting why symptom clusters, in this context, function as lived interpretive patterns that can either enable or inhibit timely escalation.

### Normalisation, uncertainty, and delayed escalation—even in hospital

Although participants were cared for in a closely monitored inpatient setting, our findings suggest that timely escalation may still be shaped not only by objective surveillance (e.g., vital signs, laboratory trends) but also by patients’ interpretive work under uncertainty. Participants sometimes normalised persistent symptoms as “part of transplant,” found it difficult to judge urgency, and drew on prior healthcare encounters when deciding whether to call for help—processes that are broadly consistent with the wider literature on symptom appraisal and help-seeking for cancer- and treatment-related complications, in which uncertainty and “wait-and-see” reasoning have been linked to delayed contact in some contexts [7]. Importantly, our findings suggest that hospitalisation does not automatically remove these barriers; rather, protective isolation may reconfigure them. Integrative review evidence indicates that isolation during HSCT is commonly experienced as disconnection from others, reduced autonomy, prolonged contemplation, and negative emotional states [17]. In such contexts, frequent symptoms and fluctuating discomfort can become “background noise,” while social and moral considerations—such as not wanting to appear demanding, not wanting to “waste” nurses’ time, or assuming staff will notice anything important—may raise the subjective threshold for reporting non-alarm concerns. This is consistent with psychosocial syntheses of cancer help-seeking showing that symptom interpretation and perceived legitimacy are shaped by anticipated judgement, role expectations, and prior experiences with services [18]. From a nursing standpoint, these dynamics provide a strong rationale for structured, routine symptom elicitation (rather than relying on patient-initiated calls), particularly during peak symptom burden in the first two post-transplant weeks; inpatient HSCT studies of electronic symptom monitoring illustrate both the feasibility of daily reporting and its potential to improve symptom assessment within transplant workflows [19].

### Perceived legitimacy and reluctance to seek help

Participants’ reluctance to seek help for “non-alarm” concerns can be interpreted through the lens of perceived legitimacy (or candidacy)—that is, how patients judge whether their concern is sufficiently warranting attention, and whether they themselves are entitled to request care at a given moment. Empirical work across health-care settings shows that worries about “wasting professionals’ time” and being seen as a “time-waster” can operate as a psychologically salient barrier to help-seeking, shaping both whether people contact clinicians and how long they delay [20,21]. Hospital contexts do not necessarily eliminate these legitimacy concerns; rather, they may be reconfigured by visible resource strain, hierarchical interactional norms, and the social work of presenting oneself as an appropriate user of care. In ethnographic work in hospital emergency care, for instance, patients’ legitimacy is actively “performed” and evaluated in interaction, and patients explicitly articulate not wanting to “bother” staff—illustrating that concerns about deservingness and burden can persist even when care is readily available on site [22]. In protective isolation during HSCT, these mechanisms may plausibly be amplified: contact is often mediated (e.g., call bells/intercoms), opportunities for informal reassurance are constrained, and symptoms are frequent and fluctuating, requiring patients to self-triage amid uncertainty about what is “expected” versus clinically important. Evidence from oncology inpatient communication research indicates that patient–nurse communication quality is shaped by organisational tempo and workload, patients’ willingness to disclose concerns, and clinicians’ capacity to detect cues and respond empathically—conditions that are highly relevant when patients fear being labelled anxious or difficult and therefore downplay symptoms or “wait to see.” [23] Taken together, this literature supports a cautious interpretation that reluctance to escalate in HSCT isolation is not simply a knowledge deficit; it may also reflect patients’ efforts to manage social risk (appearing burdensome or illegitimate) alongside clinical uncertainty, underscoring the importance of nursing practices that explicitly legitimise early reporting and reduce the interactional costs of seeking help.

### Family involvement as “remote co-management”: A family-centred care perspective

An additional finding was that physical separation during protective isolation did not remove family influence from symptom appraisal and help-seeking. Instead, family involvement was reconfigured into a form of “remote co-management”, whereby relatives participated through telephone or video communication by helping patients interpret symptoms, encouraging contact with nurses, and shaping perceptions of urgency. This finding can be understood within a family-centred care perspective, which conceptualises illness management as occurring within the patient’s family context rather than as an exclusively individual process. Family-centred care models emphasise collaboration between patients, families, and healthcare professionals, attention to family context, shared information, and education as important components of care [24].

Importantly, our findings suggest that family involvement during HSCT isolation should not be assumed to be uniformly protective. Family members sometimes lowered the threshold for help-seeking by legitimising patients’ concerns and encouraging prompt reporting, but they could also amplify uncertainty or anxiety when symptoms were interpreted through worst-case possibilities. From a family-systems perspective, such responses reflect the reciprocal influence of illness-related beliefs, communication, and coping within the family rather than a simple one-way provision of support. The Calgary Family Assessment and Intervention Models similarly emphasise family communication, beliefs, roles, and interaction patterns when understanding how families respond to illness [25].

These findings therefore extend family-centred care into the specific context of protective isolation: although relatives may be physically excluded from the bedside, they may remain active participants in patients’ appraisal and escalation decisions. For HSCT nursing practice, this suggests that, where consistent with patient preferences, family members could be included in brief education about symptom trajectories, warning patterns, and when patients should contact staff. Providing patients and families with a shared escalation framework may preserve the supportive role of family involvement while reducing anxiety driven by inconsistent or overly alarmist interpretations. Recent HSCT research likewise highlights caregiver engagement as an important component of transplant care and identifies nurses as central to supporting and coordinating that engagement [26].

### What “warning patterns” look like to patients: Trajectories, not thresholds

Clinically, education about neutropenic complications often emphasises discrete “red flags”, most notably fever, because febrile neutropenia is treated as a time-sensitive emergency and prompt evaluation and antimicrobial management are central to supportive care [27]. However, our findings suggest that—alongside these threshold-based rules—patients frequently rely on trajectory-based heuristics to decide whether a concern is escalation-worthy: worsening intensity, new or unusual combinations, or a symptom that “behaves differently than yesterday.” This is clinically plausible in HSCT because many peri-transplant toxicities are common, fluctuating, and multi-determined (e.g., mucositis, diarrhoea, nausea/vomiting, anorexia, fatigue), making simple categorical distinctions between “expected side effects” and “danger signs” difficult in practice [28,29]. In such a context, change over time (trajectory) may become more meaningful to patients than a single absolute value (threshold), particularly when routine monitoring may appear reassuring or when symptoms are frequent enough to be normalised. Importantly, several “expected” toxicities can become clinically consequential when they escalate or cluster—for example, progressive oral mucositis can impair intake and contribute to deterioration, and recent HSCT-focused evidence has linked oral mucositis with a higher likelihood of infectious complications, reinforcing why patients’ recognition of pattern change may matter for timely escalation [30]. Similarly, diarrhoea is widely recognised as one of the most common gastrointestinal complications after HSCT, and when severe or persistent it can carry hydration, electrolyte, and infectious implications, again highlighting the relevance of trajectory- and cluster-aware appraisal.

From a nursing perspective, these findings suggest that education may be strengthened by supplementing “red flag lists” with pattern language and actionable descriptors that help patients communicate change in ways clinicians can triage. Concretely, this could include teaching patients to report (i) directionality (“getting worse over the last 12–24 hours”), (ii) co-occurrence (“mouth pain now plus difficulty swallowing and reduced fluid intake”), (iii) functional impact (sleep disruption, inability to drink, orthostatic symptoms), and (iv) non-response to supportive measures (“antiemetics no longer controlling vomiting”), rather than relying solely on naming a single symptom. Such approaches are consistent with the practical logic of symptom monitoring interventions in inpatient HSCT care, where structured daily symptom reporting can surface worsening patterns earlier and reduce peak symptom burden during the first two post-transplant weeks [31]. When combined with explicit reassurance that “uncertainty is itself a reason to report,” cluster- and trajectory-informed coaching may reduce the likelihood that patients wait for a single dramatic threshold (e.g., fever) before escalating, while still preserving the urgency of fever-based rules when they occur.

### Implications for nursing practice in HSCT protective isolation

Taken together, our findings suggest several practice-relevant opportunities for HSCT nursing care in protective isolation: nurses can explicitly teach patients cluster-aware symptom interpretation and reporting (helping them describe *patterns* and *trajectories*—for example, oral pain with dysphagia and reduced intake, or diarrhoea with dizziness—rather than isolated symptoms), while simultaneously lowering the social threshold for escalation by repeatedly legitimising help-seeking (e.g., “please call even if you’re unsure”) and offering concrete examples of “non-alarm but important” concerns such as inadequate hydration, uncontrolled nausea/vomiting, or new mucosal pain that impairs intake; because spontaneous contact is often reduced in isolation, units may also benefit from routine, low-burden symptom capture (brief daily check-ins or structured symptom prompts) that routes concerning changes to nurses and normalises reporting so that escalation feels like standard care rather than “making a fuss”; finally, given that protective isolation can amplify disconnection, uncertainty, and loss of control, integrating brief psychological support and validating communication into daily nursing encounters may improve both wellbeing and timely symptom escalation, particularly when distress and physical symptoms co-evolve.

### Strengths and limitations

A strength of this study is the focus on the neutropenic phase, where symptom burden and clinical risk are both high, and where protective isolation creates a unique context for help-seeking. The analysis links symptom co-occurrence to behavioural pathways, extending existing HSCT symptom-burden research toward an actionable behavioural lens.

Several limitations warrant consideration. Findings may be shaped by the single-setting context and local care routines, potentially limiting transferability to other transplant centres. Interviews conducted during hospitalisation may reflect acute perceptions and may not capture evolving appraisal after discharge. Translation of quotations may introduce subtle meaning shifts despite cross-checking. Finally, because qualitative designs are not intended to estimate prevalence, the relative prominence of each cluster should be interpreted as thematic salience rather than frequency.

### Future research

Future studies could (i) test whether these clusters replicate across centres and transplant types using mixed-methods designs; (ii) evaluate cluster-informed education tools (e.g., “when to call” algorithms) for effects on timely reporting, distress, and safety outcomes; and (iii) develop interventions tailored to the night-time isolation context, where anxiety and insomnia appear to drive symptom withholding.

## Conclusion

During the neutropenic phase after HSCT, patients experience symptoms as interlocking clusters that shape how they interpret urgency and seek help. Fever-related constellations trigger rapid escalation consistent with clinical risk, whereas gastrointestinal/oral and sleep–anxiety constellations may be normalised, self-managed, or under-reported—particularly within protective isolation and at night. Supportive care that is explicitly cluster-informed, incorporates clear triage rules beyond fever, and legitimises emotional distress as reportable may improve both patient experience and safety during this high-risk period.

## Supporting information

S1 Checklist Consolidated Criteria for Reporting Qualitative Research (COREQ) 32-item checklist. (DOCX)

S2 File Anonymised coding hierarchy and theme-development structure. (DOCX)

S3 File De-identified qualitative excerpt matrix. (DOCX)
