# Supplementary material for: Symptom clusters and help-seeking behaviours during neutropenia after autologous haematopoietic stem cell transplantation: A qualitative study to inform oncology nursing support
Source: PLoS One. 2026 Oct 15;21(10):e0361023. doi: 10.1371/journal.pone.0361023 (PMC13671512; doi:10.1371/journal.pone.0361023)
Supplement: Supplementary file 1 [file pone.0361023.s001.docx]

**S1 Checklist. Consolidated Criteria for Reporting Qualitative Research (COREQ) 32-item checklist**

Completed against the final revised manuscript. Page numbers and section locations correspond to the final clean manuscript.

| **No.** | **Item** | **Guide question** | **Where reported / response** |
| --- | --- | --- | --- |
| 1 | Interviewer/facilitator | Which author/s conducted the interview or focus group? | Lili Jiang, RN, conducted the interviews. Methods — Research team and relationship with participants, p. 7. |
| 2 | Credentials | What were the researcher’s credentials? | Lili Jiang was a registered nurse (RN). Methods — Research team and relationship with participants, p. 7. |
| 3 | Occupation | What was the researcher’s occupation at the time of the study? | Registered nurse in the Hematology Department with clinical experience in haematology and HSCT care. Methods — Research team and relationship with participants, p. 7. |
| 4 | Gender | Was the researcher male or female? | Female. Methods — Research team and relationship with participants, p. 7. |
| 5 | Experience and training | What experience or training did the researcher have? | Eight years of clinical experience in haematology/HSCT care and structured training in qualitative research methods, semi-structured interviewing, reflexive interviewing techniques, and reflexive thematic analysis. Methods — Research team and relationship with participants, p. 7. |
| 6 | Relationship established | Was a relationship established prior to study commencement? | No established therapeutic relationship with participants before recruitment, and the interviewer was not responsible for participants’ direct clinical care. Methods — Research team and relationship with participants, p. 7. |
| 7 | Participant knowledge of the interviewer | What did participants know about the researcher? | Participants were informed of the interviewer’s professional background, role as a member of the research team, study purpose, and the voluntary nature of participation. Methods — Research team and relationship with participants, p. 7. |
| 8 | Interviewer characteristics | What characteristics were reported about the interviewer/researcher? | The interviewer’s clinical background and its potential influence on data interpretation were documented through reflexive journaling and are discussed in Methods — Researcher reflexivity, pp. 10–11. |
| 9 | Methodological orientation and theory | What methodological orientation was stated to underpin the study? | Qualitative descriptive design; data analysed using reflexive thematic analysis. Methods — Study design, p. 4; Data analysis, p. 9. |
| 10 | Sampling | How were participants selected? | Purposive maximum-variation sampling across sex, age, and post-transplant day. Methods — Participants and recruitment, pp. 5–7. |
| 11 | Method of approach | How were participants approached? | Potentially eligible patients were identified by clinical staff and subsequently approached by the research interviewer. Methods — Participants and recruitment, p. 6. |
| 12 | Sample size | How many participants were in the study? | 22 participants. Methods — Participants and recruitment, pp. 6–7; Results — Participant characteristics, p. 12. |
| 13 | Non-participation | How many people refused to participate or dropped out, and why? | Twenty-four eligible patients were approached; two declined participation because of fatigue or a preference not to participate in an interview. Twenty-two participants enrolled and completed the study, with no withdrawals after consent. Methods — Participants and recruitment, p. 6. |
| 14 | Setting of data collection | Where were the data collected? | Face-to-face interviews in participants’ protective-isolation rooms in the inpatient HSCT unit. Methods — Setting, p. 5; Data collection, p. 8. |
| 15 | Presence of non-participants | Was anyone else present besides participants and researchers? | Only the participant and the interviewer were present; no family members or clinical staff participated in the interview sessions. Methods — Data collection, p. 8. |
| 16 | Description of sample | What important characteristics of the sample were reported? | Age, sex, transplant type, diagnosis, post-transplant day, duration of neutropenia, protective isolation, febrile episode, and sleep disruption. Results — Participant characteristics and Table 1, pp. 12–13. |
| 17 | Interview guide | Were questions, prompts, or guides provided by the authors? Was it pilot tested? | The semi-structured interview guide and interview domains are described. The guide was pilot-tested with two additional eligible autologous HSCT recipients who were not included in the final analytic sample; minor wording and sequencing adjustments were made after pilot testing. Methods — Data collection, p. 8. |
| 18 | Repeat interviews | Were repeat interviews carried out? If yes, how many? | No repeat interviews were conducted. Methods — Data collection, p. 8. |
| 19 | Audio/visual recording | Did the research use audio or visual recording? | Interviews were audio-recorded with permission. Methods — Data collection, p. 8. |
| 20 | Field notes | Were field notes made during and/or after interviews? | Yes; contemporaneous field notes documented contextual features and reflexive observations. Methods — Data collection, p. 8. |
| 21 | Duration | What was the duration of the interviews? | Typically 30–60 minutes. Methods — Data collection, p. 8. |
| 22 | Data saturation | Was data saturation discussed? | The revised manuscript does not claim saturation; sample adequacy was evaluated using Malterud et al.’s Information Power framework. Methods — Participants and recruitment, pp. 6–7. |
| 23 | Transcripts returned | Were transcripts returned to participants for comment and/or correction? | No. Interview transcripts were not returned to participants for comment or correction. Methods — Rigour and trustworthiness, p. 10. |
| 24 | Number of data coders | How many data coders coded the data? | Two researchers (Lili Jiang and Junxue Hua) were involved in coding and theme development. Lili Jiang led initial coding of the complete dataset, and Junxue Hua reviewed coded extracts and emerging analytic patterns. Methods — Data analysis, p. 9. |
| 25 | Description of the coding tree | Did the authors provide a description of the coding tree? | The coding process and theme development are described in Methods — Data analysis, p. 9. A structured anonymised coding hierarchy/tree showing final theme → subtheme/analytic domain → code cluster → meaning-level codes is provided as S2 File. |
| 26 | Derivation of themes | Were themes identified in advance or derived from the data? | Meaning units were coded inductively; codes were compared and organised into code clusters, subthemes, and candidate themes and refined against the full dataset. Methods — Data analysis, p. 9. |
| 27 | Software | What software, if applicable, was used to manage the data? | NVivo version 15.3.0 (Lumivero). Methods — Data analysis, p. 9. |
| 28 | Participant checking | Did participants provide feedback on the findings? | No formal member checking was undertaken, and transcripts or final themes were not returned to participants for validation. Credibility was supported through sustained engagement with the dataset, reflexive memoing, repeated comparison, analytic discussion, and examination of disconfirming evidence. Methods — Rigour and trustworthiness, p. 10. |
| 29 | Quotations presented | Were participant quotations presented to illustrate themes/findings and identified appropriately? | Yes. De-identified participant quotations (P02, P03, etc.) are presented throughout Results, pp. 14–19; the corresponding de-identified excerpt matrix is provided as S3 File. |
| 30 | Data and findings consistent | Was there consistency between the data presented and the findings? | Representative quotations are presented alongside each analytic theme/subtheme, with a corresponding de-identified excerpt matrix in S3 File. Results, pp. 14–19. |
| 31 | Clarity of major themes | Were major themes clearly presented in the findings? | Yes. The manuscript reports three recurrent symptom clusters, four help-seeking pathways, and eight final themes. Results, pp. 13–19. |
| 32 | Clarity of minor themes | Were diverse cases or minor themes discussed? | Subthemes are reported within the eight themes; discrepant/deviant cases were actively considered during analysis. Methods — Data analysis and Rigour, pp. 9–10; Results, pp. 14–19. |
