# Supplementary material for: Symptom clusters and help-seeking behaviours during neutropenia after autologous haematopoietic stem cell transplantation: A qualitative study to inform oncology nursing support
Source: PLoS One. 2026 Oct 15;21(10):e0361023. doi: 10.1371/journal.pone.0361023 (PMC13671512; doi:10.1371/journal.pone.0361023)
Supplement: Supplementary file 2 [file pone.0361023.s002.docx]

**S2 File. Anonymised Coding Hierarchy and Theme-Development Structure**

*Supporting Information for the PLOS ONE revised manuscript*

**Coding hierarchy**

| **Final theme** | **Subtheme / analytic domain** | **Code cluster / analytic category** | **Meaning-level codes represented in the analysis** |
| --- | --- | --- | --- |
| Theme 1. A “baseline cluster” dominated everyday functioning: gastrointestinal–oral discomfort–fatigue | 1.1 A chain reaction that disrupted intake and activity | Mucosal discomfort | oral/throat discomfort; mouth sores; burning pain; dryness |
| Theme 1. A “baseline cluster” dominated everyday functioning: gastrointestinal–oral discomfort–fatigue | 1.1 A chain reaction that disrupted intake and activity | Gastrointestinal burden | nausea; taste change; early satiety |
| Theme 1. A “baseline cluster” dominated everyday functioning: gastrointestinal–oral discomfort–fatigue | 1.1 A chain reaction that disrupted intake and activity | Functional/emotional cascade | profound fatigue; reduced oral intake; weakness; low mood; symptom cascade/loop |
| Theme 1. A “baseline cluster” dominated everyday functioning: gastrointestinal–oral discomfort–fatigue | 1.2 “Expected but still unbearable”: normalisation delayed escalation | Normalisation of treatment effects | expected treatment toxicity; expected part of transplant; normalisation |
| Theme 1. A “baseline cluster” dominated everyday functioning: gastrointestinal–oral discomfort–fatigue | 1.2 “Expected but still unbearable”: normalisation delayed escalation | Endurance and delayed disclosure | endurance; delayed reporting; impaired nutrition; impaired sleep |
| Theme 1. A “baseline cluster” dominated everyday functioning: gastrointestinal–oral discomfort–fatigue | 1.3 Self-management as the first-line strategy | Low-intensity self-management | small sips; soft foods; pacing; rest; distraction; tolerating discomfort |
| Theme 1. A “baseline cluster” dominated everyday functioning: gastrointestinal–oral discomfort–fatigue | 1.3 Self-management as the first-line strategy | Escalation threshold | persistence; tighter symptom clustering; personal threshold; loss of control |
| Theme 2. The “alarm cluster” (fever–chills–pain) triggered rapid escalation and low thresholds for reporting | 2.1 Fever as a sentinel symptom | Alarm cues | fever; chills; body aches; headache; sense of danger |
| Theme 2. The “alarm cluster” (fever–chills–pain) triggered rapid escalation and low thresholds for reporting | 2.1 Fever as a sentinel symptom | Immediate action | immediate call-bell use; low threshold for reporting |
| Theme 2. The “alarm cluster” (fever–chills–pain) triggered rapid escalation and low thresholds for reporting | 2.2 Rapid escalation scripts and expectations of response | Escalation script | measure temperature; inform nurse; rapid assessment; cultures/labs; intensified monitoring |
| Theme 2. The “alarm cluster” (fever–chills–pain) triggered rapid escalation and low thresholds for reporting | 2.2 Rapid escalation scripts and expectations of response | Response-related reassurance | explanation; reassurance; reduced uncertainty |
| Theme 2. The “alarm cluster” (fever–chills–pain) triggered rapid escalation and low thresholds for reporting | 2.3 Hypervigilance after a febrile episode | Recalibrated vigilance | prior fever; heightened vigilance; feeling hot/cold; heart racing; early reporting; fear of missing warning signs |
| Theme 3. Nights amplified the sleep–anxiety–isolation cluster and reshaped help-seeking | 3.1 Night-time symptom amplification | Night-time amplification | insomnia; palpitations; rumination; loneliness; quiet environment; magnified discomfort |
| Theme 3. Nights amplified the sleep–anxiety–isolation cluster and reshaped help-seeking | 3.2 Emotional symptoms were often minimised or withheld | Low legitimacy of emotional distress | anxiety; loneliness; “not serious enough”; reluctance to disturb |
| Theme 3. Nights amplified the sleep–anxiety–isolation cluster and reshaped help-seeking | 3.2 Emotional symptoms were often minimised or withheld | Workload-sensitive withholding | perceived staff workload; withholding emotional concerns |
| Theme 3. Nights amplified the sleep–anxiety–isolation cluster and reshaped help-seeking | 3.3 Help-seeking for reassurance rather than treatment | Reassurance-seeking | need for reassurance; information; presence; uncertainty reduction; non-pharmacological support |
| Theme 4. Help-seeking was “negotiated”: appraisal, thresholds, and the need for “proof” | 4.1 Building a case before calling | Evidence accumulation | ambiguous symptoms; rechecking temperature; waiting; persistence; comparison with prior days; need for “proof” |
| Theme 4. Help-seeking was “negotiated”: appraisal, thresholds, and the need for “proof” | 4.2 Timing strategies: wait for rounds vs interrupt now | Timing negotiation | routine rounds; interruption avoidance; symptom severity; night-time exception; alarm cluster |
| Theme 5. “Watching the numbers”: lab values, monitors, and uncertainty as drivers of help-seeking | Objective indicators and uncertainty | Objective risk anchors | temperature readings; blood counts; infusion rates; “low immunity” |
| Theme 5. “Watching the numbers”: lab values, monitors, and uncertainty as drivers of help-seeking | Objective indicators and uncertainty | Interpretive uncertainty | uncertain meaning; dependence on staff interpretation; anxiety linked to numbers |
| Theme 6. Communication in isolation: practical barriers and social considerations shaped reporting | 6.1 Practical friction: PPE, distance, and “small problems” | Interactional friction | limited spontaneous contact; call bell; delayed face-to-face reassurance; isolation barriers; hesitation |
| Theme 6. Communication in isolation: practical barriers and social considerations shaped reporting | 6.2 Social calibration: not wanting to be seen as “difficult” | Social legitimacy | perceived staff workload; desire to be a “good patient”; fear of complaining; holding back |
| Theme 7. Family involvement shifted but did not disappear: “remote co-management” | Remote family influence on appraisal and help-seeking | Supportive remote co-management | phone/video contact; encouragement to report; help interpreting symptoms; comfort |
| Theme 7. Family involvement shifted but did not disappear: “remote co-management” | Remote family influence on appraisal and help-seeking | Anxiety-amplifying remote influence | worst-case framing; increased anxiety; night-time influence |
| Theme 8. What participants wanted: concrete guidance to support timely and appropriate help-seeking | 8.1 Clear escalation criteria | Actionable escalation rules | immediate-report rules; observation versus escalation; uncertainty reduction |
| Theme 8. What participants wanted: concrete guidance to support timely and appropriate help-seeking | 8.2 A night-time plan | Night-time coping/escalation plan | night-time reassurance; insomnia; panic; coping plan |
| Theme 8. What participants wanted: concrete guidance to support timely and appropriate help-seeking | 8.3 Symptom-cluster framing rather than single-symptom advice | Cluster-based guidance | symptoms as a package; cluster-based advice; combined symptom interpretation |
| Theme 8. What participants wanted: concrete guidance to support timely and appropriate help-seeking | 8.4 Permission to report emotional distress without feeling “unreasonable” | Legitimising emotional help-seeking | anxiety as reportable; legitimising help-seeking; earlier disclosure |

Theme-development note. The final analysis comprised three recurrent symptom clusters (gastrointestinal–oral discomfort–fatigue; fever–chills–pain; sleep disruption–anxiety–isolation), four broad help-seeking pathways (immediate reporting, watchful waiting, self-management followed by reporting, and minimisation/withholding), and eight final themes. S3 File provides the de-identified excerpt matrix linking representative quotations to the corresponding analytic domains.
