# Supplementary material for: Symptom clusters and help-seeking behaviours during neutropenia after autologous haematopoietic stem cell transplantation: A qualitative study to inform oncology nursing support
Source: PLoS One. 2026 Oct 15;21(10):e0361023. doi: 10.1371/journal.pone.0361023 (PMC13671512; doi:10.1371/journal.pone.0361023)
Supplement: Supplementary file 3 [file pone.0361023.s003.docx]

**S3 File. De-identified Qualitative Excerpt Matrix**

*Representative excerpts supporting the thematic analysis*

| **Participant code** | **Theme** | **Subtheme / domain** | **De-identified excerpt reported in manuscript** | **Analytic relevance** |
| --- | --- | --- | --- | --- |
| P07 | Theme 1 | 1.1 Chain reaction disrupting intake and activity | “When my mouth hurts, I don’t want to eat; when I don’t eat, I feel weak; when I’m weak, everything feels worse—including the nausea. It’s like a loop.” | Illustrates the self-reinforcing interaction between oral discomfort, reduced intake, weakness, and nausea. |
| P14 | Theme 1 | 1.2 Normalisation delayed escalation | “I thought this is part of transplant, so I tried to endure. I only called when I realised I had taken almost nothing for the whole day.” | Shows how classifying symptoms as expected reduced the perceived legitimacy/urgency of reporting despite functional impact. |
| P09 | Theme 1 | 1.3 Self-management as first-line strategy | “At first I tried to manage it myself—small sips, resting. But when the diarrhoea and nausea came together, I felt I couldn’t control it anymore.” | Marks the transition from self-management to reporting when symptoms clustered and controllability declined. |
| P03 | Theme 2 | 2.1 Fever as a sentinel symptom | “Once the temperature goes up, it’s not a small thing in this period. I press the call bell immediately.” | Demonstrates fever as an unambiguous high-risk cue prompting immediate escalation. |
| P19 | Theme 2 | 2.2 Rapid escalation scripts | “When they come quickly and explain what they’re checking, I feel I’m not alone with the fear.” | Shows how prompt clinical response and explanation reduce uncertainty and provide reassurance. |
| P05 | Theme 2 | 2.3 Hypervigilance after fever | “After I had fever once, even a small chill makes me nervous. I’m afraid I’ll miss the early sign.” | Illustrates how prior fever lowers subsequent reporting thresholds and increases vigilance. |
| P11 | Theme 3 | 3.1 Night-time symptom amplification | “In the daytime you can distract yourself. At night it’s quiet, and your mind keeps turning. Even small discomfort feels bigger.” | Shows how night-time quiet and reduced distraction amplify both bodily and psychological distress. |
| P02 | Theme 3 | 3.2 Emotional symptoms minimised or withheld | “I didn’t want to disturb them for ‘just anxiety’. But it kept me awake and made everything feel worse.” | Illustrates the lower perceived legitimacy of emotional distress and reluctance to burden staff. |
| P08 | Theme 3 | 3.3 Reassurance-seeking | “I called not because I needed medicine, but because I needed someone to tell me whether this is normal.” | Shows help-seeking aimed at interpretation and reassurance rather than treatment. |
| P16 | Theme 4 | 4.1 Building a case before calling | “I observe first. If it passes quickly, maybe it’s nothing. If it repeats, then I feel I have a reason to ask.” | Demonstrates the need to accumulate evidence before considering a concern legitimate enough to report. |
| P20 | Theme 4 | 4.2 Timing strategies | “If it’s not fever, I wait for rounds. But if it’s night and I feel something is wrong, I won’t wait.” | Shows context-sensitive timing of help-seeking based on alarm status, night-time uncertainty, and interruption concerns. |
| P06 | Theme 5 | Watching the numbers | “They tell me my neutrophils are very low, but I don’t know what symptoms are ‘danger’ and which ones are ‘normal’. That uncertainty is stressful.” | Illustrates the gap between awareness of objective risk markers and confidence in symptom interpretation. |
| P12 | Theme 5 | Watching the numbers | “If someone explains what today’s results mean, I feel calmer and I don’t overthink every sensation.” | Shows reliance on professional interpretation of laboratory/monitoring information to regulate uncertainty. |
| P10 | Theme 6 | 6.1 Practical friction in isolation | “In isolation you can’t just ask casually. You have to press the bell. For small symptoms, I hesitate.” | Shows how the mechanics of isolation increase the effort and social threshold required for minor symptom disclosure. |
| P17 | Theme 6 | 6.2 Social calibration | “I see they are busy. I don’t want them to think I complain too much, so I hold back unless it’s serious.” | Demonstrates withholding shaped by perceived staff workload and concern about being seen as demanding. |
| P04 | Theme 7 | Remote co-management by family | “My family tells me to call the nurse immediately. Sometimes that helps me act, but sometimes it makes me more scared.” | Shows the dual role of family contact in facilitating escalation while also amplifying anxiety. |
| P13 | Theme 8 | 8.1 Clear escalation criteria | “If they give a simple rule—what to report immediately—I would feel less uncertain.” | Supports the need for explicit, actionable escalation rules. |
| P21 | Theme 8 | 8.2 Night-time plan | “Nights are hardest. If there was a plan for what to do when you can’t sleep and feel panic, it would help.” | Supports a structured night-time coping and escalation plan. |
| P07 | Theme 8 | 8.3 Symptom-cluster framing | “It’s not one symptom. It comes as a package. Advice should match that.” | Directly supports cluster-aware symptom education rather than single-symptom advice. |
| P02 | Theme 8 | 8.4 Permission to report emotional distress | “If someone told me it’s okay to ask for help even for anxiety, I would not wait until it becomes unbearable.” | Supports explicit legitimisation of emotional symptoms as appropriate reasons to seek help. |

**Scope note.** Only excerpts already reported in the submitted manuscript are included here. This file is not a substitute for the complete interview transcripts and does not disclose unreported participant-level clinical details.
